# Identification of an Alu‐repeat‐mediated deletion of *OPTN* upstream region in a patient with a complex ocular phenotype

**DOI:** 10.1002/mgg3.159

**Published:** 2015-06-02

**Authors:** Kala F. Schilter, Linda M. Reis, Elena A. Sorokina, Elena V. Semina

**Affiliations:** ^1^ Department of Pediatrics and Children's Research InstituteMedical College of WisconsinMilwaukeeWisconsin53226; ^2^ Department of Cell Biology, Neurobiology and AnatomyMedical College of WisconsinMilwaukeeWisconsin53226

**Keywords:** Alu‐mediated recombination, anterior segment dysgenesis, *CCDC3*, *OPTN*

## Abstract

Genetic causes of ocular conditions remain largely unknown. To reveal the molecular basis for a congenital ocular phenotype associated with glaucoma we performed whole‐exome sequencing (WES) and whole‐genome copy number analyses of patient DNA. WES did not identify a causative variant. Copy number variation analysis identified a deletion of 10p13 in the patient and his unaffected father; the deletion breakpoint contained a single 37‐bp sequence that is normally present in two distinct Alu repeats separated by ~181 kb. The deletion removed part of the upstream region of optineurin (*OPTN*) as well as the upstream sequence and two coding exons of coiled‐coil domain containing 3 (*CCDC3*); analysis of the patient's second allele showed normal *OPTN* and *CCDC3* sequences. Studies of zebrafish orthologs identified expression in the developing eye for both genes. *OPTN* is a known factor in dominant adult‐onset glaucoma and Amyotrophic Lateral Sclerosis (ALS). The deletion eliminates 98 kb of the *OPTN* upstream sequence leaving only ~1 kb of the proximal promoter region. Comparison of transcriptional activation capability of the 3 kb normal and the rearranged del(10)(p13) *OPTN* promoter sequences demonstrated a statistically significant decrease for the deleted allele; sequence analysis of the entire deleted region identified multiple conserved elements with possible cis‐regulatory activity. Additional screening of *CCDC3* indicated that heterozygous loss‐of‐function alleles are unlikely to cause congenital ocular disease. In summary, we report the first regulatory region deletion involving *OPTN*, caused by Alu‐mediated nonallelic homologous recombination and possibly contributing to the patient's ocular phenotype. In addition, our data indicate that Alu‐mediated rearrangements of the *OPTN* upstream region may represent a new source of affected alleles in human conditions. Evaluation of the upstream *OPTN* sequences in additional ocular and ALS patients may help to determine the role of this region, if any, in human disease.

## Background

Anterior segment dysgenesis of the eye results from abnormal embryonic development and is often associated with congenital or early‐onset glaucoma (Idrees et al. 2006). Pediatric glaucoma is difficult to manage with most affected individuals requiring surgical intervention; some degree of vision loss is commonly observed (Khitri et al. 2012). Anterior segment dysgenesis and pediatric glaucoma are highly heterogeneous conditions. While a number of genes have been found to play a role in these disorders, including *CYP1B1* (MIM: 601771), *LTBP2* (MIM: 602091), *MYOC* (MIM: 601652), *FOXC1* (MIM: 601090), *PAX6* (MIM: 607108), and *PITX2* (MIM: 601542) (Khan 2011; Reis and Semina 2011), many cases are still awaiting molecular diagnosis.

Copy number variation (CNV) includes deletions or duplications of various sizes that are identified in comparison to a reference genome and can be inherited or occur de novo (Feuk et al. 2006). Three major mechanisms for CNV in the human genome include nonallelic homologous recombination typically mediated by low‐copy repeats or highly homologous repetitive sequences like Alu and LINE, nonhomologous end‐joining, and the Fork Stalling and Template Switching models (Gu et al. 2008). CNV alleles were found to be associated with both dominant and, when coupled with another deleterious mutation of a gene within the region, recessive phenotypes (Lesnik Oberstein et al. 2006; Pieras et al. 2011). It is widely accepted that CNVs, especially deletion alleles, often result in affected phenotypes or increase an individual's susceptibility for the disease (Feuk et al. 2006; Beckmann et al. 2007; Stranger et al. 2007; Liu et al. 2011a). A surprisingly extensive variability in copy number has been identified in the human genome and was proposed to represent a major source of inter‐individual genetic diversity, possibly underlying the incomplete penetrance and variable expressivity of many inherited Mendelian disorders as well as variation in phenotypic expression associated with more complex disease (Beckmann et al. 2007). Copy number variation analysis was instrumental to the discoveries of regulatory regions/mutations associated with disease phenotypes (Lauderdale et al. 2000; Volkmann et al. 2011).

In this paper, we present identification and characterization of a deletion involving the *OPTN* (MIM: 602432) and *CCDC3* (NM_031455.3) genes in a patient with a congenital ocular phenotype involving glaucoma.

## Methods

### Human subjects

The human study was approved by the Institutional Review Board of the Children's Hospital of Wisconsin and informed consent was obtained from every subject and/or legal guardian, as appropriate.

### Copy number variation and WES analyses

Copy number variation analysis via Affymetrix Genome‐Wide Human SNP Array 6.0 (Santa Clara, CA) was undertaken as previously described with custom region analysis for RefSeq genes (NCBI build GRCh37/hg19) and 203 genes known to be involved in ocular development including 200 kb of potential regulatory regions (Schilter et al. 2013). The Database of Genomic Variants (http://projects.tcag.ca/variation/) was used as a control population along with 30 unaffected in‐house controls; a control population specifically matched to the patient's Trinidad and Tobago ancestry was not available. Predesigned and custom‐designed TaqMan probes (Life Technologies, Carlsbad, CA) (Table S1) were utilized for independent verification/confirmation of the copy number states. Amplification of the deleted allele and sequencing of the deletion breakpoints was performed using the primers indicated in Table S2. The resultant product was cloned into pCRII TOPO vector (Life Technologies) and sequenced.

Whole‐exome sequencing was undertaken through Perkin Elmer, Inc (Branford, CT) using Agilent Sure Select v4+UTR for exome capture and analyzed as previously described (Reis et al. 2013). Data were evaluated for mutations in 203 genes known to be involved in ocular development (Schilter et al. 2013) through the Geospiza GeneSifter Analysis program hosted through Perkin Elmer Bioinformatics. The entire exome was analyzed using the SNP & Variation Suite (SVS; Golden Helix, Bozeman, MT) as previously described (Deml et al. 2014; Weh et al. 2014).

### Gene sequencing

Complete sequence for the coding regions of the *OPTN* (NM_001008211.1) and *CCDC3* (NM_031455.3) genes was obtained for the proband utilizing the primers indicated in Table S2. Sequencing of *CCDC3* was undertaken in an additional 115 patients affected with ocular disorders and 183 (90 Caucasian, 93 Hispanic) controls. Sequences were reviewed manually and using Mutation Surveyor (SoftGenetics, State College, PA). All mutations were confirmed by independent sequencing reactions using new PCR products. Variants of interest were reviewed for their frequency in dbSNP (http://www.ncbi.nlm.nih.gov/projects/SNP/), Exome Variant Server (EVS; http://evs.gs.washington.edu/EVS/), and ExAC Browser (http://exac.broadinstitute.org).

### Expression studies in zebrafish

All experiments were executed in agreement with the guidelines described by the Institutional Animal Care and Use Committee at the Medical College of Wisconsin. The zebrafish *optn* (NM_001100066.2), *ccdc3a* (chromosome 4; NM_001025510), and *ccdc3b* (chromosome 11; NM_001020570) constructs were generated using the PCR products obtained with the primers indicated in Table S2. In situ hybridization was performed as previously described (Liu and Semina 2012).

### Reported constructs and luciferase assays

*OPTN* wild‐type promoter sequence was amplified with the primers indicated in Table S2 to generate a 3049‐bp product. This fragment was first inserted into a pCRII‐TOPO plasmid and then subcloned into a pGL4.10[*luc2*] vector (Promega, Madison, WI) using *Kpn*I (located within the pCRII polylinker) and AvrII (located within *OPTN* exon 1 at +74 bp of transcriptional start site) restriction sites for the insert and *Kpn*I/*Nhe*I digest for the *pGL4.10[luc2]* vector, thus resulting in a 2923‐bp *OPTN* wild‐type promoter (−2849 to +74) luciferase reporter construct, *WT(OPTN* _2923)*luc*. To generate an *OPTN* promoter construct corresponding to the del(10p13) allele, the pCRII‐TOPO plasmid containing the 2113‐bp deleted allele's sequence was subjected to partial digest with *Kpn*I and *Xho*I; the 1762‐bp deletion fragment was cloned into the corresponding sites of the *pGL4.10[luc2]* vector. Additional inserted sequence, corresponding to −945 to +74 of the *OPTN* region that is shared between wild‐type and mutant alleles, was excised from the wild‐type construct and added to the deletion constructs using a *Xho*I site (located at −945 from *OPTN* transcription site); this resulted in the 2781‐bp sequence corresponding to the patient's deleted allele (1592 bp of *CCDC3* intron 2 followed by −1115 to +74 fragment of *OPTN* promoter), *Patient(CCDC3:1592/OPTN:1189)luc*. A third construct containing the portion of promoter region that is shared between wild‐type and mutant alleles was generated using an adaptor DNA fragment created with the following modified oligonucleotides: 5′‐[Phos]CTATTGGCCAGGCTGGTCTCG‐3′ and 5′‐[Phos]AGTTCGAGACCAGCCTGGCCAATAGGTAC‐3′. The oligonucleotides were annealed to create a double stranded fragment with *Kpn*I and Eco31I sticky ends. The *Patient(CCDC3:1592/OPTN:1189)luc* plasmid was digested with *Kpn*I/Eco31I and the excised fragment was exchanged with the adaptor fragment resulting in a 1238‐bp proximal promoter construct (−1164 to +74), *WT(OPTN:1238)luc*. All constructs were verified by sequencing.

Luciferase reporter assays were performed as previously described (Sorokina et al. 2011). Briefly, human embryonic kidney cells (293HEK) in 24‐well plates were transfected utilizing equimolar amounts of *WT(OPTN* _2923)*luc* (100 ng), *Patient(CCDC3:1592/OPTN:1189)luc* (98 ng), *WT(OPTN:1238)luc* (77 ng), and pGL4.10[*luc2*] empty vector (59 ng), as well as 60 ng of *pcDNA3.1_LacZ* (*β*‐galactosidase‐containing reference plasmid) for internal control of transfection efficiency; cell confluence at transfection was at 80–90%. To account for variation in the amount of DNA, empty *pcDNA3.1* was added to ensure all transfections contained the same total amount of DNA. Transfection was facilitated by Lipofectamine 2000 (Life Technologies) which was utilized according to the manufacturer's protocol. All experiments were repeated at least four times in quadruplicates. The 293HEK cells were selected for these experiments because of their high transfection efficiency as well as previously demonstrated endogenous OPTN expression and utility in OPTN studies (Anborgh et al. 2005; Morton et al. 2008).

## Results

### Clinical features of affected patient

A patient diagnosed with anterior segment dysgenesis and glaucoma and his unaffected parents were enrolled into the study. At 2 months of age, the patient demonstrated faintly opaque corneas, shallow (not fully formed) anterior chambers, hypoplastic irides, and possible retrolental masses in both eyes (poorly visualized). Ultrasound identified severe bilateral retinal detachments. Exam under anesthesia at 3 months of age revealed progression of the corneal opacity such that no discernable structures were visible behind the cornea. The corneas were reported to be oval in shape (right cornea 9 mm wide and 6 mm high; left 10 mm wide and 8 mm high) with diffuse clouding; cystic epithelia and greater stromal changes were present centrally. Elevated intraocular pressures (34–38 mmHg) were noted in the left eye while right eye pressures were low (9, 7, and 10 mmHg); tactile assessment confirmed firm left eye and soft right eye. The overall appearance of the eye was suggestive of major disorganization, dysgenesis, and dysplasia. Electroretinography (ERG) responses were indiscernible, consistent with retinal detachments. The patient has no other health or developmental concerns; his hearing was tested and determined to be normal. The patient's father is of East Indian/Caucasian ancestry from Trinidad and Tobago and has normal vision with no ocular anomalies reported. The patient's mother is of mixed Caucasian ancestry, has mild myopia (OD 20/40, OS 20/20) and astigmatism, and was noted to have a single CHRPE (Congenital Hypertrophy of the Retinal Pigment Epithelium) on her left retina. No additional relatives were available for testing. Prior screening by Sanger sequencing excluded mutations in *PITX2*,* FOXC1*,* BMP4*,* CYP1B1*,* FOXE3*, and *NDP* (Reis et al. 2011, 2012, and unpublished data).

### Whole‐genome copy number variation and exome sequencing analyses

**Figure 1:**
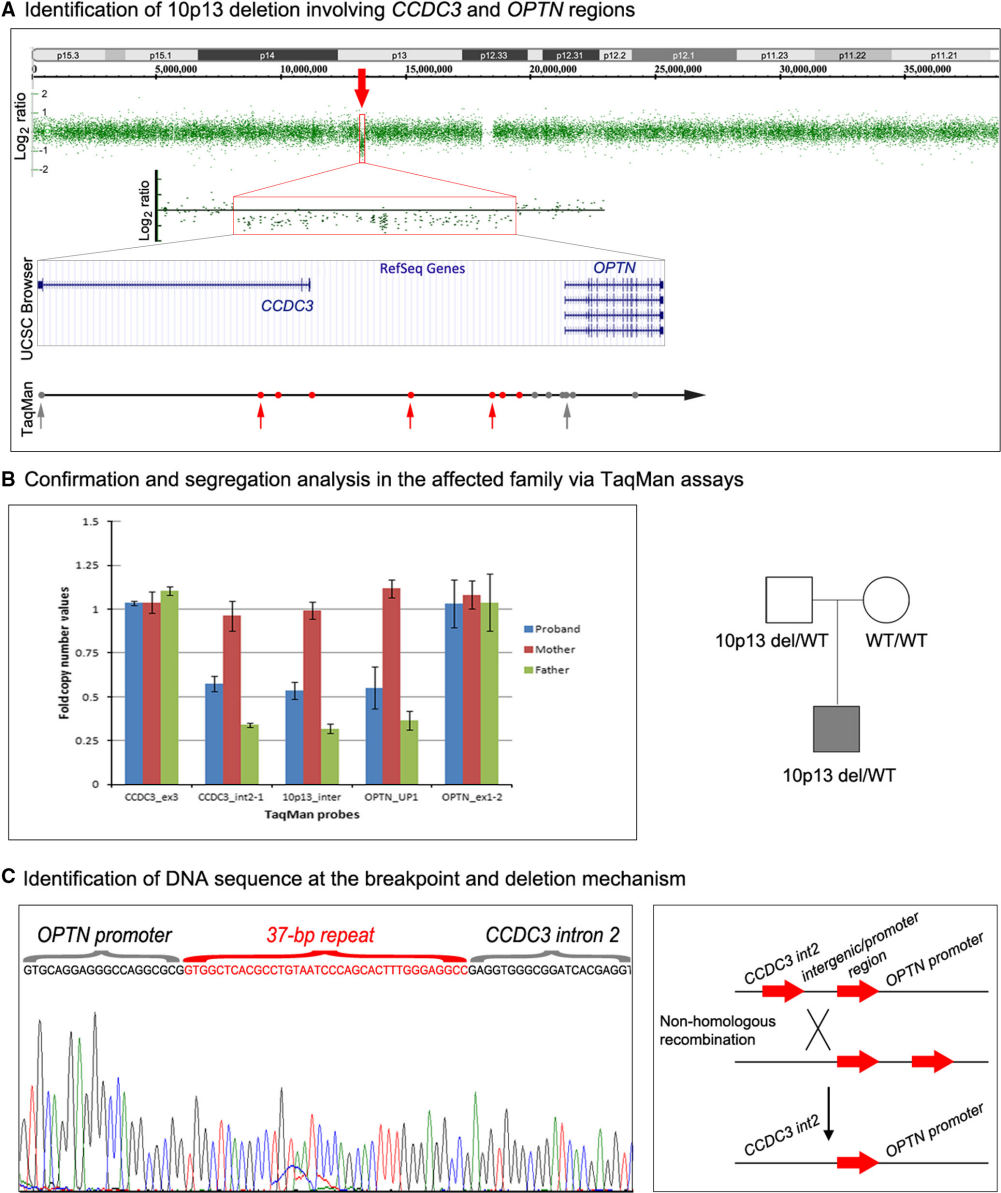
Identification and characterization of del(10)(p13) involving the *CCDC3 and OPTN* gene regions. (A) Affymetrix Genotyping Console view showing the deletion of the 10p13 region identified in Patient 1 (red arrow); the entire chromosome 10 results are shown as well as an enlargement of the deleted area. The UCSC Genome Browser (http://genome.ucsc.edu) view of the deleted region indicating the positions of genes is included; positions of TaqMan probes (Table S1) are indicated with gray (diploid status) and red (haploid status) circles; positions of assays included in (b) are indicated with arrows. (B) Results of copy number analysis via TaqMan assays for Patient 1 as well as his mother and father; deletion status is also noted on the pedigree shown on the right. (C) DNA sequence across breakpoint for the deleted allele. Sequences of the *OPTN* promoter, the 37‐repeat and *CCDC3* intron 2 are indicated; reference sequences NM_001008211.1 (*OPTN)* and NM_031455.3 (*CCDC3*) were used. The nonhomologous recombination mechanism likely involved in the generation of the deleted allele is depicted in a schematic drawing on the right.

The patient's DNA was analyzed using WES and whole‐genome CNV analysis. Analysis of WES data excluded pathogenic mutations in currently known genes associated with ocular development. Review of rare/novel variants throughout the exome also failed to identify any likely pathogenic variants to explain the ocular findings. By CNV analysis, the patient was found to have a deletion of at least 178 kb at 10p13 (Fig. 1); the deletion was not present in the Database of Genomic Variants or in‐house controls. The deletion encompasses 203 markers unanimously calling a copy number state of 1 and spans from the second intron of the *coiled‐coil domain containing 3* (*CCDC3*) gene to the 5′ end of the *optineurin* (*OPTN*) gene (Fig. 1A). Additional qPCR analysis with TaqMan probes (Table S1) confirmed the haploid state of the deleted area including the first two exons of *CCDC3* and the diploid state of the 3rd exon of *CCDC3* and all exons of *OPTN*; the deletion breakpoint was mapped to the region located between 1 and 1.5 kb upstream of the *OPTN* gene. Analysis of parental samples demonstrated that the unaffected father of the patient also carried the deletion and the unaffected mother had normal copy number for the entire region (Fig. 1B). No additional CNVs of interest were identified.

Primers located in proximity to the identified deletion boundaries were used to amplify a 2113‐bp fragment from the deletion allele. The sequencing revealed a 37‐bp repeat at the site of the deletion (Fig. 1C); in the wild‐type allele, the repeat sequence is located 1078 bp upstream of exon 1 of *OPTN* and 18,768 bp upstream of exon 3 of *CCDC3* (within intron 2) with a 181,457‐bp region separating these two repeats. The deleted allele retained one of the 37‐bp repeated sequences, the other repeat was deleted along with the ~181‐kb sequence between the repeated elements (Fig. 1C). BLAST analysis of the 37‐bp sequence determined this region to be part of an Alu repeat; specifically, the Alu elements surrounding the deletion belong to the Yk4 and Sx4 inter‐Alu subfamilies (http://www.repeatmasker.org) that share 85% overall homology and 100% identity for the 37‐bp element. The 181 kb deleted region contains multiple Alu elements: more than 90 regions with 75–92% similarity to the repeats located at the breakpoints were found (with the 37‐bp sequence being present in five of these regions). The exact coordinates for the deletion were determined as chr10:12959447 (within *CCDC3* intron 2) and chr10:13140967 (within *OPTN* upstream region). Sequencing of the *CCDC3* and *OPTN* genes in the patient identified no changes in the *CCDC3* gene and a common SNP variant in the *OPTN* gene, c.553‐5C>T (rs2244380; allele frequency 82% based on EVS), ruling out contribution of a second intragenic mutation at this loci to his phenotype.

### Expression studies in zebrafish

In situ hybridization analyses of zebrafish orthologs of the human *OPTN* and *CCDC3* genes were performed to evaluate their potential role in embryonic eye development. Zebrafish *optn* was detected in the developing head and eye in 24‐ to 120 hpf (hours post fertilization) embryos (Fig. S1). Expression of *optn* in the anterior segment of the eye was first observed at 48 hpf and continued to be strongly present in the developing iris and periocular mesenchyme in 120‐hpf embryos.

One zebrafish *ccdc3* ortholog has previously been reported (NM_001025510; zebrafish chromosome 4); using BLAST analysis, we identified a second ortholog located on zebrafish chromosome 11 (NM_001020570) and designated these genes *ccdc3a* and *ccdc3b*, respectively. The ccdc3a and ccdc3b proteins exhibit high amino acid conservation to the human CCDC3 protein, 67% and 62%, respectively. In situ hybridization studies revealed a dynamic expression pattern in zebrafish embryos from 24‐ to 120 hpf (Fig. S2). The expression of *ccdc3a* and *ccdc3b* at 18–24 hpf is broad with strong signals observed in the developing head, eyes, and somites. At later stages (48‐ through 120‐hpf) *ccdc3a* becomes enriched in the region of the developing anterior segment of the eye, particularly its ventral domain, with continued broad expression in the head; *ccdc3b* becomes strongly expressed in the developing pharyngeal arches, brain, and the hyaloid vasculature in the eye (Fig. S2).

### Screening of additional human patients

CNV data from 40 patients affected with different forms of anterior segment dysgenesis and/or glaucoma were examined for the presence of 10p13 deletions but no additional alleles were identified.

Sequencing of *CCDC3* in 115 patients affected with ocular disorders identified one common SNP (c.549 + 31A>G in intron 2 (rs112487830)) and two frameshift variants in the *CCDC3* gene, both likely resulting in complete loss‐of‐function alleles. The first change, c.459_460dupGT, p.(Phe154Cysfs*69), was seen in a Hispanic patient with syndromic anterior segment dysgenesis with congenital glaucoma and her unaffected mother. This change has not been reported in any control individuals including 183 samples screened by us, nor 13,006/121,406 control alleles available in EVS/ExAC. The other change, c.463delT, p.(Ser155Leufs*67), was found in a South Asian proband with cataract‐microcornea syndrome; however, the variant was not present in the proband's affected father and cousin. The c.463delT, p.(Ser155Leufs*67) allele was not present in 183 control individuals screened by us nor 13,006 EVS alleles but was reported in 25/16510 (0.2%) South Asian alleles in the ExAC Browser. Based on these data, we concluded that heterozygous loss‐of‐function mutations in *CCDC3* are unlikely to result in ocular disease and thus the *CCDC3* deletion observed in our patient is unlikely to explain the ocular phenotype.

### Analysis of transcriptional activities of OPTN wild‐type and mutant promoters

**Figure 2:**
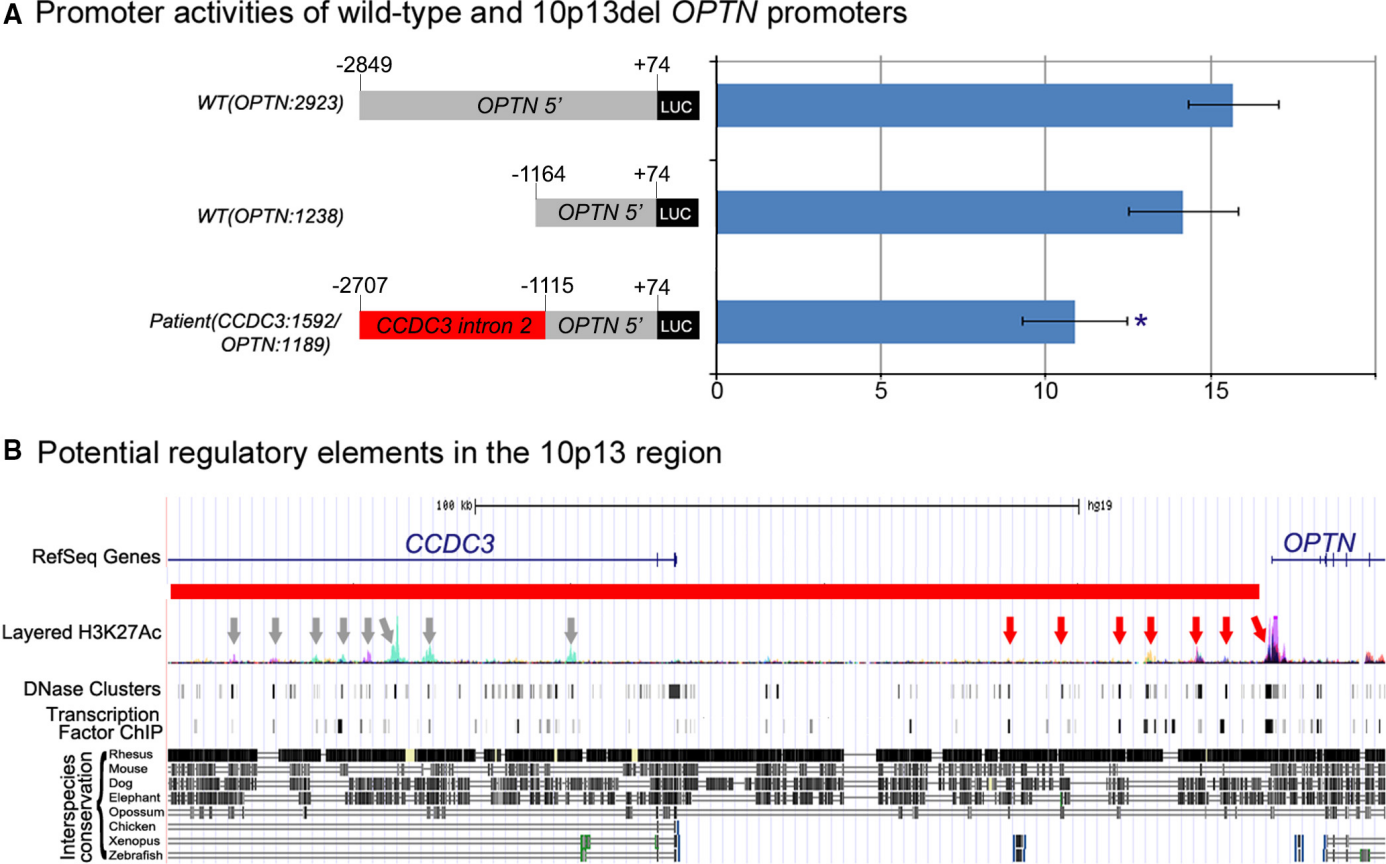
Analysis of the effect of the deletion on the *OPTN* promoter. (A) Promoter activities of *WT(OPTN:2923)luc*,*WT(OPTN:1238)luc*, and *Patient(CCDC3:1592/OPTN:1189)luc* reporters. Student paired *t*‐test with a one‐tailed distribution was utilized to compare values. The promoter activity of *Patient(CCDC3:1592/OPTN:1189)luc* demonstrated a significant decrease (marked with asterisk [*]) in comparison to experiments performed with wild‐type *WT(OPTN:2923)luc* reporter (*P* < 0.001) or wild‐type *WT(OPTN:1238)luc* reporter (*P* < 0.013). Sequences corresponding to the *OPTN* and *CCDC3* genes are marked, with coordinates indicated in respect to the *OPTN* transcriptional start site. (B) Schematic of genomic region encompassing the deletion with potential distant regulatory elements indicated. Genome Browser view is shown with ENCODE project data for H3K27Ac marks (that are frequently located near active regulatory elements) on seven cells lines, digital DNase I hypersensitivity clusters in 125 cell types, transcription factor ChIP‐seq data and multiple interspecies sequence alignments highlighting regions of strong homology (often associated with conserved regulatory elements). Arrows indicate potential regulatory elements as proposed by several of the above mentioned assays; red arrows point to elements located in the intergenic space closer to the *OPTN* gene while gray arrows mark indicates elements positioned within the *CCDC3* gene.

The *OPTN* and *CCDC3* genes are located in a head‐to‐head orientation with ~98‐kb distance between their 5′UTRs. Previously published studies of the *OPTN* promoter involved analysis of a 1077‐bp fragment corresponding to the (−856 to +221) region upstream of *OPTN* (Sudhakar et al. 2009). This sequence remained intact in Patient 1 while the region immediately upstream of this sequence was deleted (Fig. 1). To explore the effects of the deletion on the function of the *OPTN* promoter, we studied transcriptional activities associated with three different luciferase reporter constructs in human embryonic kidney cells (293HEK) as previously described (Sorokina et al. 2011): the first construct, *WT(OPTN*:2923)*luc*, encompasses the −2849 to +74 region of the wild‐type *OPTN* promoter; the second reporter, *WT(OPTN:1238)luc*, contains the −1164 to +74 region of the wild‐type *OPTN* promoter; and the third construct, *Patient(CCDC3:1592/OPTN:1189)luc*, corresponds to the deleted allele identified in the affected patient and includes 1592 bp of *CCDC3* intron 2 sequence followed by the (−1115 to +74) fragment of the *OPTN* promoter (Fig. 2A). Transfection of wild‐type constructs *WT(OPTN*:2923)*luc* or *WT(OPTN:1238)luc* into human embryonic kidney (HEK293) cells resulted in ~16‐ and 14‐folds activation, respectively, in comparison to the promoterless reporter vector; transfection of the mutant, *Patient(CCDC3:1592/OPTN:1189)luc*, reporter yielded ~11‐fold activation, signifying a statistically significant decrease in the transactivation activity of this promoter (*P* < 0.001 when compared to *WT(OPTN:2923)* and *P* < 0.013 when compared to *WT(OPTN:1238)*) (Fig. 2A). Cumulatively, these data suggest that the (−1164 to −2849) region of the *OPTN* promoter contains positive regulatory elements since the reporter *WT(OPTN:1238)luc* that lacks this region yielded ~90% of the activity of the entire region; in addition to this, fusion of the *CCDC3* intronic sequence with the *OPTN* promoter (as occurred in the affected patient) resulted in a statistically significant downregulation of luciferase activity to 69% of wild type, consistent with an inhibitory effect of the *CCDC3* region on the transcriptional activities of the immediate *OPTN* promoter.

Additionally, analysis of the 181‐kb deleted region using UCSC Genome Browser (http://genome.ucsc.edu/) revealed multiple *DNase*I hypersensitivity clusters, open states of chromatin, histone modification sites, and transcription binding sites determined by the ENCODE project (ENCODE Project Consortium 2012) (Fig. 2B). The sites of particular interest are located at ~0.5, 8, 12, 20, 26, 34, and 42 kb upstream of *OPTN* exon 1; these regions were identified in multiple assays and six out of seven were found to be highly conserved in multiple species (Fig. 2B). All of these regions, except for the most proximal upstream element, were removed by the deletion in the mutant allele. Since these elements may be involved in transcriptional regulation of *OPTN*, their absence in the deleted allele as well as a change in chromatin context (for the most proximal site) are likely to affect the level and/or sites of *OPTN* expression.

## Discussion

In this report, we present the identification of the first deletion involving the regulatory region of the *OPTN* gene along with two coding exons of the *CCDC3* gene, del(10)(p13), in a patient with congenital ocular disease.

The *OPTN* gene encodes a cytosolic protein with three coiled‐coil domains, a zinc finger domain and two additional motifs (Wild et al. 2011). *OPTN* was first linked to human disease through linkage analysis in a family affected with normal tension open angle glaucoma followed by the identification of multiple heterozygous mutations in different adult‐onset glaucoma patients (Rezaie et al. 2002). Since then, substantial variability in the pathogenicity of *OPTN* variants in different populations has been reported with only a few variants showing consistent association with glaucoma in different ethnic groups (Ayala‐Lugo et al. 2007). In addition to this, heterozygous and homozygous *OPTN* mutations have been identified in patients affected with familial, middle‐age‐onset, Amyotrophic Lateral Sclerosis (ALS), a progressive disorder characterized by degeneration of motor neurons of the primary motor cortex (Maruyama et al. 2010; Maruyama and Kawakami 2013), including one study which identified a risk allele for *OPTN*‐associated glaucoma in combination with a missense change in another ALS‐associated gene, raising the possibility of digenic inheritance (Weishaupt et al. 2013). Recent studies of the effects of *OPTN* mutations have suggested a dominant‐negative or gain‐of‐function effect for many of the characterized disease‐associated variants (Shen et al. 2014; Turturro et al. 2014).

*OPTN* transcripts were detected in the trabecular meshwork, nonpigmented ciliary epithelium, retina, and other tissues in humans (Rezaie et al. 2002; Liu et al. 2011b). *Optn* was found to be strongly expressed in the embryonic mouse eyes (Rezaie et al. 2007), which is consistent with our zebrafish data and indicates a possible role for *OPTN/optn* in ocular development. Optineurin is involved in many cellular functions including protein trafficking, secretion, cell division, and antiviral/antibacterial signaling (Kachaner et al. 2012; Ying and Yue 2012). The identified deletion of the *OPTN* upstream region is likely to have multiple effects on gene expression. In the reporter assays, the ~3‐kb fragment of the patient's allele encompassing the telomeric deletion breakpoint (and carrying the mixture of the *OPTN* promoter and the *CCDC3* intronic sequences) showed a reduced transcriptional activity in comparison to the corresponding normal *OPTN* upstream sequence, supporting a possible impact on the *OPTN* promoter activity through the changes in its immediate environment. We also demonstrated the presence of multiple conserved sequences in the larger deleted area; these regions are likely to serve as distal cis‐regulatory elements, such as enhancers, silencers, and insulators (Sakabe et al. 2012; Nelson and Wardle 2013) and thus their removal, as well as introduction of foreign sequence from the internal *CCDC3* region, may have produced additional impacts on *OPTN* expression. Misexpression of genes due to changes in their regulatory landscape is becoming associated with a growing number of human disorders (Lauderdale et al. 2000; Volkmann et al. 2011; Spielmann et al. 2012). Considering the broad roles of *OPTN/optn* discussed above, its embryonic misexpression may have diverse negative effects on development of ocular structures. Therefore, further studies of the deleted region and encompassed sequences using transgenic and/or genome editing approaches in animal models or cell culture would be required. The presence of the 10p13 deletion in the unaffected father may be explained by incomplete penetrance and modification of its phenotypic expression by additional genetic/environmental factors; alternatively, this deletion may represent a benign variant, possibly unique to the patient's Trinidad and Tobago ancestry. Evaluation of the *OPTN* upstream region in ocular and ALS patients can help to determine the role of this region, if any, in human disease.

The mechanism of the identified 181‐kb deletion appears to be nonhomologous recombination facilitated by misalignment of two Alu repeats containing a 37‐bp region of identical sequence and 85% overall homology. Alu‐mediated nonhomologous recombination events are a major cause of CNV and disease (Deininger and Batzer 1999). The region encompassing the coding exons of the *OPTN* gene has been previously found to have a high density of Alu repeats that predisposes *OPTN* to Alu‐mediated coding region deletions (Iida et al. 2012); in the above mentioned study, authors investigated deletions within the *OPTN* coding region in patients affected with ALS and found different types of deletions occurring due to Alu‐mediated recombination (Iida et al. 2012). In this paper, we present evidence that the upstream region of *OPTN* is also enriched in Alu repeats and prone to nonhomologous recombination events. Therefore, this region needs to be carefully examined in glaucoma and ALS patients to identify/rule out possible rearrangements that may contribute to the observed phenotypes.

*CCDC3* (also termed Favine; fat/vessel‐derived secretory protein), another gene disrupted by the identified deletion, encodes for a secretory factor that was isolated from adipocytes and endothelial cells of mouse aorta and adipose tissue (Kobayashi et al. 2010). The function of the CCDC3 protein is yet unknown with no known phenotype/disease association in humans or animals. The contribution of the *CCDC3* deletion to the patient's ocular phenotype is currently unclear. While expression studies in zebrafish indicate a possible function in ocular development, the presented human studies suggest no causative role for *CCDC3* heterozygous loss‐of‐function alleles in congenital ocular phenotypes; further investigation of the gene is needed to determine whether disruption of *CCDC3* in other phenotypes and/or by different mechanisms (i.e., dominant negative, complete loss of function, digenic) may be a factor in ocular disease.

## Conflict of Interest

None declared.

## Supporting information


**Figure S1**. Expression studies of *optn* in zebrafish embryos. In situ hybridization using wild‐type embryos at 24, 48, and 120 hpf is shown. ase, anterior segment of the eye; b, brain; e, eye; i, developing iris; sm, skeletal muscles; pom, periocular mesenchyme.

**Figure S2.** *ccdc3a* (**A**) and *ccdc3b* (**B**) in situ hybridization in zebrafish embryos at 18‐, 24‐, 48‐, 72‐, and 120‐hpf. ase, anterior segment of the eye; b, brain; e, eye; hv, hyaloid vasculature; oc, oral cavity; pa, pharyngeal arches; s, somites; sm, skeletal muscles.

**Table S1.** Summary of TaqMan assays utilized in this study.

**Table S2.** PCR primers and conditions for amplification of gene sequences under study.
