# Supplementary figures and images for: Identification of an Alu‐repeat‐mediated deletion of OPTN upstream region in a patient with a complex ocular phenotype
Source: Mol Genet Genomic Med. 2015 Jun 2;3(6):490–9. doi: 10.1002/mgg3.159 (PMC4694134; doi:10.1002/mgg3.159)

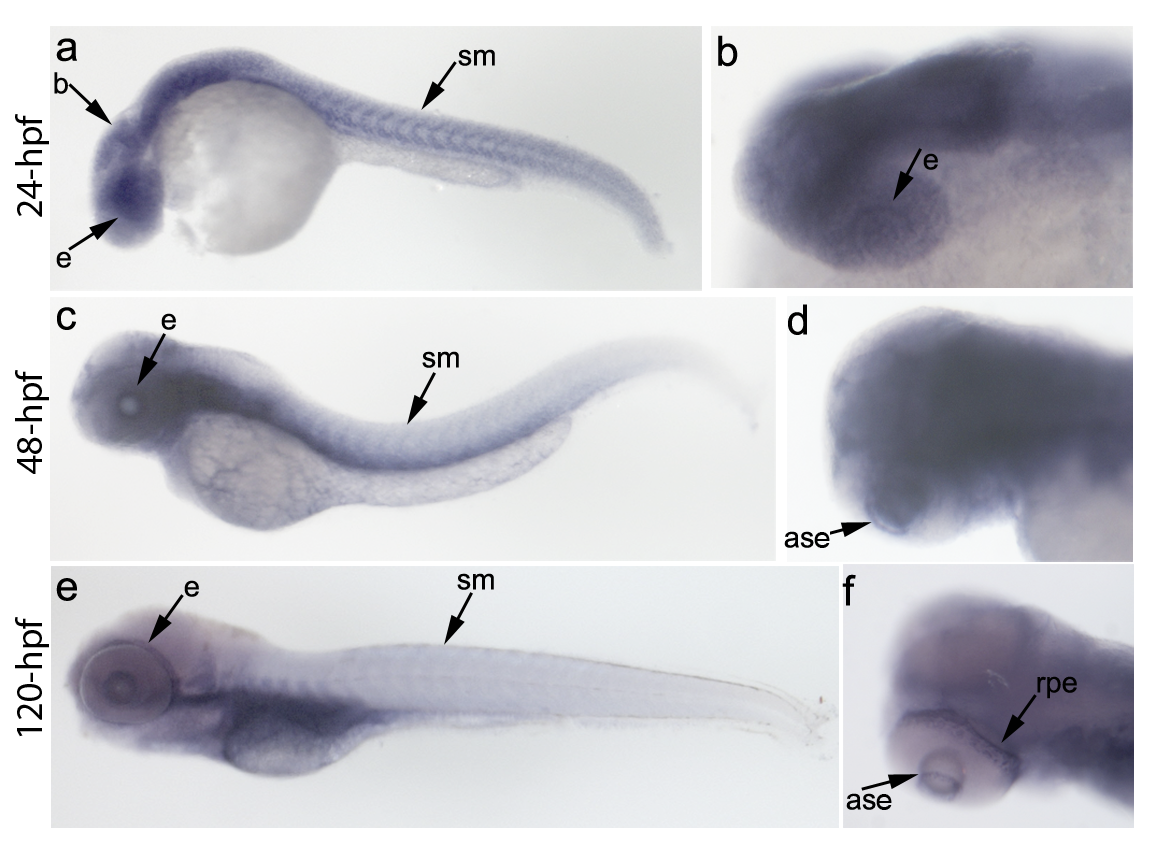

Supplement: Supplementary file 1 — Figure S1. Expression studies of optn in zebrafish embryos. In situ hybridization using wild‐type embryos at 24, 48, and 120 hpf is shown. ase, anterior segment of the eye; b, brain; e, eye; i, developing iris; sm, skeletal muscles; pom, periocular mesenchyme. [file MGG3-3-490-s001.tif]

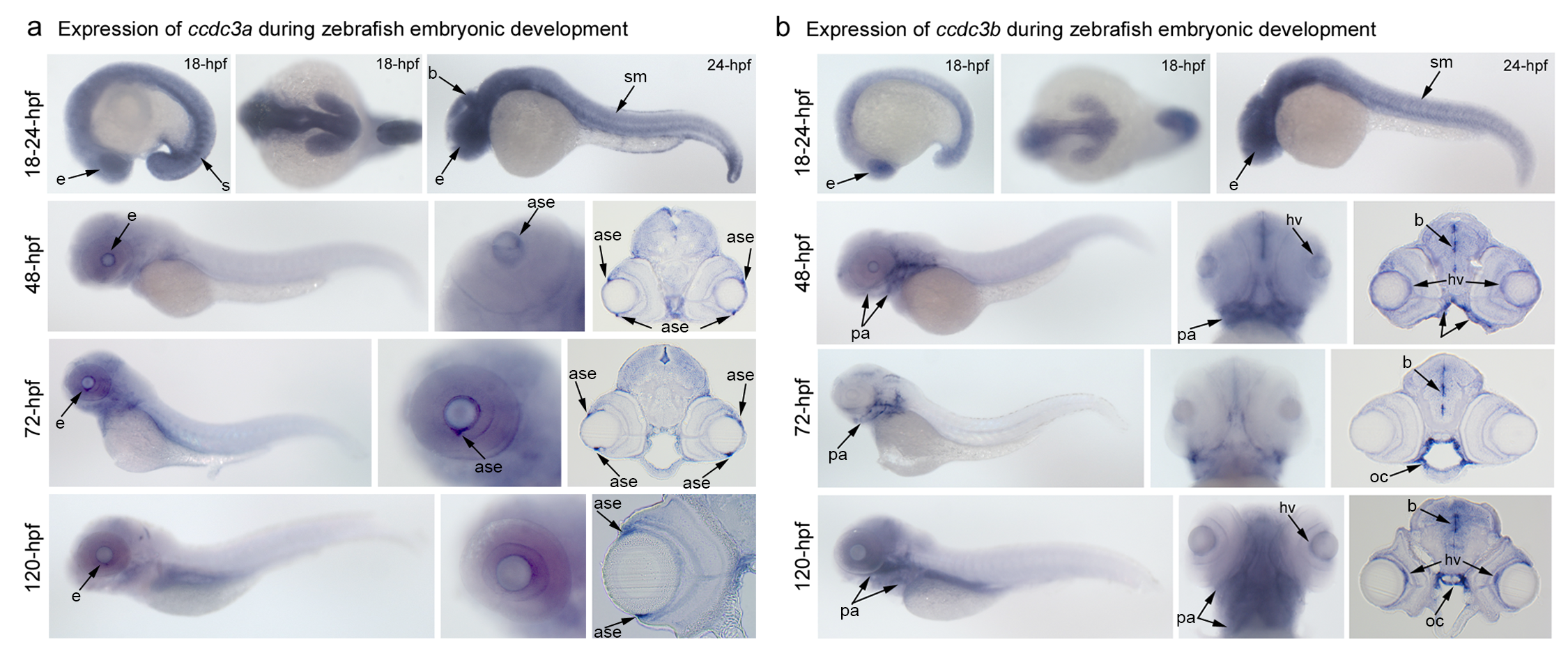

Supplement: Supplementary file 2 — Figure S2. ccdc3a (A) and ccdc3b (B) in situ hybridization in zebrafish embryos at 18‐, 24‐, 48‐, 72‐, and 120‐hpf. ase, anterior segment of the eye; b, brain; e, eye; hv, hyaloid vasculature; oc, oral cavity; pa, pharyngeal arches; s, somites; sm, skeletal muscles. [file MGG3-3-490-s002.tif]
