# Supplementary material for: Identification of an Alu‐repeat‐mediated deletion of OPTN upstream region in a patient with a complex ocular phenotype
Source: Mol Genet Genomic Med. 2015 Jun 2;3(6):490–9. doi: 10.1002/mgg3.159 (PMC4694134; doi:10.1002/mgg3.159)
Supplement: Supplementary file 3 — Table S1. Summary of TaqMan assays utilized in this study. [file MGG3-3-490-s003.doc]

| **Probe** | | **Cytogenetic band** | **Genome coordinate** | **Location** |
| --- | --- | --- | --- | --- |
| **Study name** | **Applied Biosystems ID** |
| *CCDC3_ex3* | Hs01964536_cn | 10p13 | chr10:12940571 | *CCDC3*; exon 3 |
| *CCDC3_int2-1* | Hs05211993_cn | 10p13 | chr10:12960182 | *CCDC3*; intron 2 |
| *CCDC3_int2-2* | Hs05123658_cn | 10p13 | chr10:12962497 | *CCDC3*; intron 2 |
| *CCDC3_ex1* | Hs02266506_cn | 10p13 | chr10:13043199 | *CCDC3*; exon 1 |
| *10p13_inter* | Hs05094983_cn | 10p13 | chr10:13091804 | Intergenic: 50,278-bp upstream of *OPTN* exon 1; 48,100-bp upstream of *CCDC3* exon 1 |
| *OPTN_UP1* | Custom design | 10p13 | chr10:13136128 | *OPTN*; 5,931-bp upstream of exon 1 |
| *OPTN_UP2* | Custom design | 10p13 | chr10:13140173 | *OPTN*; 1,890-bp upstream of exon 1 |
| *OPTN_UP3* | Custom design | 10p13 | chr10:13140599 | *OPTN*; 1,464-bp upstream of exon 1 |
| *OPTN_UP4* | Custom design | 10p13 | chr10:13141127 | *OPTN*; 936-bp upstream of exon 1 |
| *OPTN_UP5* | Custom design | 10p13 | chr10:13141675 | *OPTN*; 394-bp upstream of exon 1 |
| *OPTN_ex1-1* | Custom design | 10p13 | chr10:13142172 | *OPTN*; 5' end of exon 1 |
| *OPTN_ex1-2* | Custom design | 10p13 | chr10:13142274 | *OPTN*; 3' end of exon 1 |
| *OPTN_int1* | Hs05102844_cn | 10p13 | chr10:13143810 | *OPTN*; intron 1, 1495-bp from exon 1 |
| *OPTN_ex10* | Hs00466721_cn | 10p13 | chr10:13165999 | *OPTN;* exon 10 |
