# Supplementary material for: Identification of an Alu‐repeat‐mediated deletion of OPTN upstream region in a patient with a complex ocular phenotype
Source: Mol Genet Genomic Med. 2015 Jun 2;3(6):490–9. doi: 10.1002/mgg3.159 (PMC4694134; doi:10.1002/mgg3.159)
Supplement: Supplementary file 4 — Table S2. PCR primers and conditions for amplification of gene sequences under study. [file MGG3-3-490-s004.doc]

| **Gene/region** | **Set#** | **Region** | **Forward primer** | **Reverse primer** | **PCR product size** |
| --- | --- | --- | --- | --- | --- |
| **Human sequencing** | | | | | |
| 10p13 Deletion |  |  | TGGAGCCTGAGTCCATGAAG (CCDC3 intron 2) | CTATGTGCATCCAGAGTACC (OPTN upstream region) | 2113 |
| *CCDC3* | 1 | exon 1 | GGGACCCTTTTAAAGCGTGG | TTCTCACTTGACACCGACAG | 669 |
| *CCDC3* | 2 | exon 2 | CCATGCCAGACATTGATGTG | CGCTAATACTCTCTGATCTG | 414 |
| *CCDC3* | 3 | exon 3 | ATGTCACCGTCAAGGACATC | ACCCTTGAAATAGCTGCATG | 512 |
| *OPTN* | 1 | exon 2 | TATGTCCACATGGATGCCTC | GTAGACATGGGCAAGGTATC | 494 |
| *OPTN* | 2 | exons 3&4 | ATGCACACATGCGCGTGCA | CAGCTACCACCTATGGAAAC | 512 |
| *OPTN* | 3 | exon 5 | CAAGGCTAAGCATGGCATCT | GATCTAGGAGTCTAGACACG | 413 |
| *OPTN* | 4 | exon 6 | TCCCAGAGCTCTGCGATTAA | CACTGGAATTTCCTCAATCC | 386 |
| *OPTN* | 5 | exon 7 | GTGCACATCTGAATGTTTGG | AGGTCACAACATTTGACCTC | 301 |
| *OPTN* | 6 | exon 8 | CAGCTGTGCTTGTTCACTAG | ACAGTGGTTGCACAATCCTG | 439 |
| *OPTN* | 7 | exon 9 | GTCTGTGAATCAATTCTAGCC | TTGACACAGAGCAGGACAAG | 469 |
| *OPTN* | 8 | exon 10 | CACCAGCCAGTCTTAATTGG | TGCTCACACATTAACTGGAAC | 397 |
| *OPTN* | 9 | exon 11 | TATGGCCAGGTCTAGTGAAG | TCTGCTAGGACTCCTTCAGA | 452 |
| *OPTN* | 10 | exon 12 | CTACTGGAGTGTTCAGAAGG | GAAGTTACAAACCCTAGATGC | 342 |
| *OPTN* | 11 | exon 13 | CTCAATTCTAGGCATGAGCC | AGATCCACTGAGCACTTTCC | 460 |
| *OPTN* | 12 | exon 14 | TCACAAGGGCTATTGAAGGA | GCGCGAACACAGCTATTCTT | 347 |
| *OPTN* | 13 | exon 15 | ATGAACCTTGGCAGTGTAGTT | TAATGAGACTGACGGGTGCT | 336 |
| *OPTN* | 14 | exon 16 | GTGCATCGTGATGACTTCAG | TTTGGTAGAGACAGGGTTCC | 541 |
| **Zebrafish expression constructs** | | | | | |
| *optn* |  |  | catggcatctggatcatcga | GGATCTGGTAAGGTTACTAG | 1605 |
| *ccdc3a* |  |  | CATGCACCTTGCGCTTCTGC | AGCTCACAGTCCATGTGTGT | 835 |
| *ccdc3b* |  |  | AGAGGCTGTTTGCAAAGCTG | CCAGCTCTTAGTTCTTGAGC | 849 |
| **Reporter construct** | | | | | |
| *OPTN* upstream region |  |  | CATCTACCCATGGAAGTGTG | CTCAGGGTCACTGTTTCCTC | 3049 |
